# Institutional Review Board Obligations Regarding Study Funding Sufficiency

**DOI:** 10.1002/eahr.70008

**Published:** 2026-03-05

**Authors:** Holly Fernandez Lynch, Tasneem Mohammad

**Affiliations:** ^1^ Associate professor of medical ethics in the Department of Medical Ethics and Health Policy at the Perelman School of Medicine at the University of Pennsylvania; ^2^ JD/MBE candidate at the Carey Law School at the University of Pennsylvania

**Keywords:** institutional review board (IRB), study budget, funding, early termination

## Abstract

Given their obligations to ensure ethical research, we argue that institutional review boards (IRBs) bear a responsibility to minimize the possibility that proposed clinical studies will terminate early for insufficient funding. Underfunded studies raise several ethical concerns, including potentially failing to satisfy the social value requirement for research, the possibility of leaving study participants worse off, and exposing participants to the “completion misconception,” thereby impairing adequate informed consent. Although the practice is not currently typical, IRBs should confirm funding sufficiency before approving a study, ideally working with institutional offices or other experts skilled in budget review. In most cases, IRBs should withhold approval from studies lacking full funding at the point of review. In exceptional cases, however, an IRB may approve an important but underfunded study if the researcher discloses funding insufficiency and termination risk to prospective participants, does not leave participants worse off, has a clear funding plan, and follows checkpoints for securing funding within a reasonable timeframe. Even with IRB attention to study budgets at the point of approval, subsequent events outside an IRB's control may cause a study to lose its originally promised funding. To minimize these cases and their impact, IRBs can encourage institutions to buttress their funding agreements, seek sponsor certifications, require notification of material funding changes, and support investigators through contingency planning and communication with participants.

BrainStorm Cell Therapeutics, Inc. is a biotechnology company developing an investigational product called NurOwn, an autologous cell therapy intended for the treatment of amyotrophic lateral sclerosis (ALS).^1^ BrainStorm has already sought and failed to secure US Food and Drug Administration (FDA) approval for NurOwn as a treatment for ALS based on a phase 3 clinical trial that missed its prespecified endpoints and raised safety concerns.^2^ Despite these results, the company filed a biologics license application for NurOwn over the FDA's protest.^3^ Following a negative advisory committee vote, BrainStorm withdrew its application in October 2023,^4^ but has not abandoned the drug, instead announcing plans for a second phase 3 trial to further assess NurOwn's efficacy.

BrainStorm's co‐CEO stated in March 2024 that the company would likely raise funds for their latest trial in “stages” instead of waiting to start the trial until they obtained all funding necessary to complete it.^5^ On later investor calls, BrainStorm was vague about whether it had reconsidered this plan to launch the study prior to having all the funds in hand. In December 2024, the company stated its intention to begin dosing trial participants in the first quarter of 2025, even though it was still exploring funding sources,^6^ and in November 2025, BrainStorm reported that it was “making steady progress toward stabilizing our financial situation and initiating our Phase 3b study of NurOwn,” which the FDA issued clearance to initiate.^7^, ^8^ As of this writing, there has been no public disclosure that BrainStorm's trial was initiated, let alone reviewed by an institutional review board (IRB); there are 15 anticipated sites listed on the study's ClinicalTrials.gov posting, which indicates that the study is not yet recruiting.^9^ Given current practices, however, it is important to consider whether an IRB would even be aware that a proposed study under its review lacks sufficient funding for completion. Moreover, if the IRB did become aware, how would it respond?

Starting a trial without the financial means to finish it raises serious ethical concerns. A study that is not completed will be unable to answer its scientific question, which can substantially diminish or negate the study's social value. Yet it is social value—the potential to answer a research question for the benefit of future patients—that differentiates research from clinical care and justifies exposing participants to study burdens and risks, including visits, tests, procedures, and exposures to an investigational product.^10^ Ending a trial due to lack of financial resources would breach reasonable participant expectations of study completion and waste researcher and funder resources that could be put to better use.^11^ In addition, studies that lack adequate resources may be unable to provide proper care to participants who experience adverse events or other concerns.^12^ In sum, there is at least a default ethical expectation that IRBs should not allow under‐resourced trials—perhaps like BrainStorm's upcoming study of NurOwn—to begin.

IRBs serve an integral role in promoting ethical clinical research, but their oversight practices and authority may have significant gaps when it comes to addressing the ethical implications of study budgets. At present, IRBs are not regularly obtaining funding information from sponsors or researchers proposing new clinical studies. We argue that this is a mistake. IRBs have a responsibility to consider the adequacy of study funding when evaluating the risks and benefits of proposed research. To fulfill this responsibility, IRBs should request information on funding status as part of the requirements for IRB approval. In most cases, IRBs should withhold approval until full study funding is secured. However, in exceptional cases, it may be ethically permissible for an IRB to allow an underfunded study to proceed with additional safeguards, including firm plans for future funding and disclosure of funding gaps to participants. Although the arguments presented here are specific to clinical research, we expect broader applicability to other types as well, likely in proportion to burden, risk, and expected social value.

## ETHICAL CONCERNS REGARDING STUDY TERMINATION FOR FINANCIAL REASONS

A clinical study is complete if researchers enroll, complete data collection for, and analyze data from enough participants to answer the research question. Empirical research has examined reasons why clinical studies fail, including poor participant accrual, safety or efficacy concerns, and funding issues.^13^ One recent study found that 5.6% of adult interventional cancer trials registered on ClinicalTrials.gov failed to complete due to “inadequate budget,”^14^ suggesting a substantial problem. Trials that end early due to safety or efficacy concerns and those that are closer to completion when terminated can still generate valuable information about the investigational product or other intervention.^15^ However, terminating a study early for financial reasons raises several ethical concerns.^13^

First, financial termination can impede satisfaction of the social value requirement for ethical research. A clinical study has social value if the knowledge gained can broadly lead to improvements in health and well‐being or increase knowledge.^10^ A social value requirement ensures that researchers do not expose participants to potential harms without the possibility that these sacrifices will be outweighed by scientific benefit.^16^ For a clinical study to have adequate social value, it must have adequate statistical power, i.e., enroll enough participants, and collect enough data about those participants for a long enough period to answer the research question. If a study is underfunded, it may fail on each of these elements—enrollment, data collection, and duration—resulting in a waste of resources, including institutional or sponsor funding, equipment, treatments, and time.^17^

Second, participants could be left worse off for having participated in a study that terminates early due to insufficient funding. For example, if a participant receives an incomplete course of treatment, they may not only miss out on potential benefits of that study treatment but also risk being precluded from enrollment in future trials exploring novel treatments because of potential confounding variables associated with prior exposure.^17^ In addition, clinical studies provide ongoing monitoring and care to participants to ensure that they are properly tapered off experimental treatment and treated for possible side effects or adverse reactions. If a study terminates early, participants could be abruptly cut off from both the product and continuing medical services,^17^ for example, risking withdrawal symptoms and other adverse events, especially in studies examining treatments for substance abuse disorder or other mental illness. Participants in studies of infectious disease treatments could also develop resistance if the intervention is halted early. For these reasons, the Participant Follow‐up Improvement in Research Studies and Trials (Participant FIRST) Work Group, which formed after several Alzheimer's disease trials were abruptly terminated for futility in 2018 and 2019, recommends that clinical trials should have procedures in place for the orderly closeout of participants should a trial end early, including adequate funding and resources allocated for closeout in the initial study budget.^18^ Underfunded studies, however, are not likely to be able to meet this ethical standard.

Third, launching an underfunded study could impair the adequacy of participant informed consent. Consent materials often already contain disclosure language indicating that researchers or sponsors reserve the right to terminate the trial “at any time for any reason.”^17(p.306)^ However, this language is not detailed enough in describing the risk of noncompletion that an underfunded study could face. Indeed, it would be reasonable for participants to assume, even after reading a generic clause on termination in a consent form, that their study has all the necessary financial resources to be completed, leading to a type of “completion misconception.”^19^ Surprising participants with the early termination of a study due to underfunding without explicitly disclosing that this was a possibility violates the ethical obligation to disclose all material study information to prospective participants.^16^ Adequate study funding and expected completion are material because they are likely to shape the decisions of prospective participants about enrollment, especially for those motivated by altruistic goals of advancing science for the benefit of others.^19^ As Wertheimer notes, the importance of disclosing the possibility of—and possible reasons for—study noncompletion may be even greater than the importance of disclosing information about personal risks and benefits, since participants will likely seek information about the latter on their own but may not consider the former.^19^

## CURRENT IRB APPROACHES TO ADDRESSING FUNDING SUFFICIENCY

Given the clear ethical importance of adequate study funding—and because IRBs are responsible for protecting participants by ensuring that research meets ethical requirements, including avoidance of unnecessary

**Underfunded studies will have questionable social value because of the high risk of noncompletion; IRBs should adopt a default rule of withholding approval until adequate funding is secured.**

risks and burdens—it seems to fall clearly within an IRB's responsibility to take an active role in assessing whether there is enough funding for a proposed study to be completed.^20^ Are IRBs currently meeting this responsibility, and what impediments might prevent them from doing so?

Little has been written about IRB reviews of study funding sufficiency. Our anecdotal discussions with several IRB leaders suggest that most institutions do not have consistent procedures for an IRB to carry out this task. A more formal review of publicly available IRB materials leads to the same conclusion. After examining protocol and consent form templates, human research policies and procedures, guidance for researchers, frequently asked questions, and other IRB submission‐related resources from a random sample of 20 of the 100 research institutions with the most NIH funding in FY24, we found that only two sites (10%) mention adequacy of funding as a factor that the institution's IRB would consider when deciding whether to approve a proposed biomedical research study. If these top‐tier institutions, which are likely some of the best resourced with the most sophisticated policies, usually are not assessing funding as part of their IRB review process, it is unlikely that other institutions are doing so.

The two sites we identified where IRBs are reviewing study resources demonstrate why and how this could be done, as well as potential shortcomings. The University of Southern California states in its Human Research Protection Program (HRPP) Policy Manual that the “assurance of adequate financial resources for proposed research is inherent in the HRPP's responsibilities and obligations to human participants” because participants “may be put at risk if the protocol cannot be carried out as approved, due to inadequate resources.”^21(p.15)^ The manual also states that the IRB in most cases will “rely on access to budget information” available in its online system during review of proposed studies.^21^ Similar language appears in Michigan State University's HRPP Manual, which requires the IRB to “review applications to determine if each research study has the necessary resources to protect subjects’ rights and welfare,” including “monetary and non‐monetary resources.”^22^ These sites demonstrate important steps toward the approach we have in mind, demonstrating that IRBs—or the HRPPs of which they are a part—can help ensure that proposed studies have appropriate funding. Importantly, however, both sites focus on the financial and other resources needed to minimize risk and not explicitly on those resources needed for study completion. While both are important, the latter is what is most critical to address the social value and completion misconception concerns noted above.

It is possible that an institutional office or entity other than the IRB may be reviewing funding sufficiency, such as clinical trial offices or grant and contract offices, as we learned anecdotally sometimes occurs. Because IRBs may not themselves be well suited to evaluate whether a study's budget is sufficient, as a matter of expertise, time, and resources, it would be wise for them to share this responsibility with other entities likely to be more skilled at budget review. Nonetheless, IRBs must be confident that *someone* has evaluated the budget of a proposed study and deemed it sufficient to avoid the ethical concerns associated with studies that cannot finish. Importantly, since the goal is study completion, and not just proper care for participants following early termination, it is essential to consider funding sufficiency not only on an annual basis or site‐by‐site, but overall. Commercial IRBs or single IRBs responsible for reviewing multisite research are likely especially well‐positioned to take this overall perspective.

## IDENTIFYING FUNDING INSUFFICIENCY

**Figure 1:**
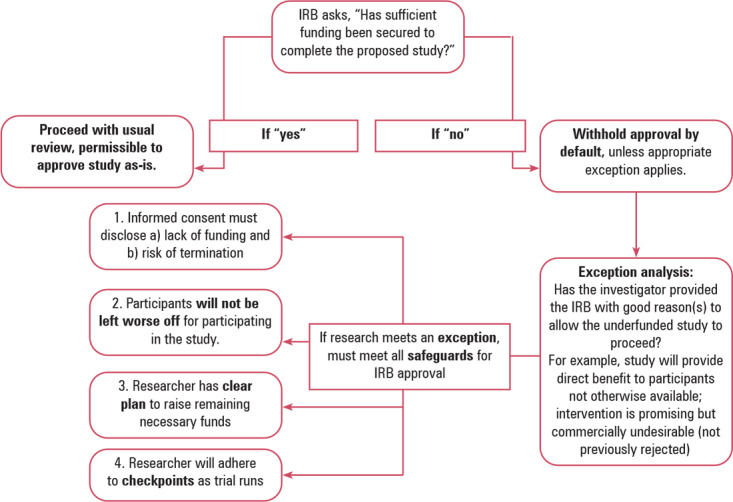
IRB Review of a Proposed Study's Funding Sufficiency

IRBs have an ethical obligation to evaluate if the funding available at the time of review is adequate to accomplish the goals of the study. They can meet this obligation through their own direct review, assuming they have adequate internal capacity, or by building into their workflow a step that procures confirmation from appropriate institutional offices or experts that the study budget is both adequate and the funds are assured to be available when needed (see figure 1).

To begin the process, IRBs might ask on a protocol submission form whether the researcher has secured the funds necessary to complete the study. Checking “yes” would indicate that they (or the overall study principal investigator) have all necessary funding in hand, or at least a reliable promise that the necessary funding will be available for the duration of the study. For example, apart from the recent widespread cancellation of federal research grants under President Trump's second administration (discussed below), if the researcher has secured a federal grant that will be disbursed over time, proof of grant funding should generally be deemed sufficiently reliable for the IRB's purposes. Similarly, if the sponsor is a private company, the researcher could be asked to provide attestation from the sponsor that it has adequate funds set aside to cover the stated budget.

If, on the other hand, the researcher or sponsor indicates that full funding has not yet been secured to complete the study, this should trigger additional IRB review. The researcher could then be required to answer a series of follow‐up questions, such as what percentage of needed funds has been secured, the plan for obtaining remaining funding, and when the rest of the funding is expected to become available. This information would inform the IRB about whether to withhold approval until the study is fully funded or allow it to proceed with safeguards.

## RESPONDING TO FUNDING INSUFFICIENCY

Underfunded studies will have questionable social value because of the high risk of noncompletion. As a result, IRBs should adopt a default rule of withholding approval until adequate funding is secured. However, there are potentially exceptional circumstances under which IRBs may allow an underfunded study to proceed if it can comply with required safeguards. For example, one exception may arise if a proposed study could provide direct benefit to participants who have no good alternatives, such that refusing IRB approval could inhibit access to important benefits that might accrue to individual participants even if the study is not completed. Another exception may arise for studies that are scientifically sound but unable to secure funding due to commercial constraints, such as studies involving interventions for rare or ultra‐rare diseases, or due to the nature of competitive grant funding, such as studies based on novel or disfavored but reasonable scientific theories.^23^ Importantly, this exception should not apply to studies that remain inadequately funded because prior studies indicate that the intervention lacks promise.

Given the varied reasons that could justify proceeding with insufficient funding, it is difficult to offer more specific guidance regarding the universe of acceptable exceptions; IRBs will need to make case‐by‐case determinations. However, the critical point is that the burden of proof lies with the investigator to convince the IRB why their study should be allowed to proceed despite insufficient funding. They may provide peer‐reviewed literature, letters of support from experts, grant scores and comments, or any other appropriate context to buttress their case. IRBs may also decide to call on outside experts to assess the legitimacy of the investigator's rationale, when necessary.

If sufficiently convinced, the IRB may consider approval without full funding, so long as several safeguards are met. First, the IRB should require researchers to explicitly disclose the funding shortfall and its potential consequences during the consent process.^17^ The informed consent form should state that the study does not currently, at the time of enrolling participants, have all the funding needed for it to be completed, so there is a strong possibility that the study may terminate prematurely and, potentially, abruptly. Steps should be in place to ensure that this material information is adequately understood by participants before accepting their enrollment, such as asking a teach‐back question at the end of the consent process on this specific point.

Second, the IRB should require researchers to demonstrate that participants would not be worse off if the study is in fact ended early. For example, before enrollment begins, the IRB should be certain of adequate funding for orderly termination for enrolled participants, should it become necessary. The study also must have adequate funding in hand to provide enrolled participants necessary medical care for adverse events, on‐going monitoring, and follow‐up care.

Third, the IRB should condition approval on the presentation of a compelling plan for how the sponsor and researcher will obtain the remaining necessary funding. For example, an underfunded sponsor may have scheduled upcoming meetings with interested investors, a planned merger or buyout with another company, or a debt financing proposal in place to secure the remaining funding. As outlined above, IRBs and institutions can decide together what evidence would be acceptable. Case‐by‐case consideration is appropriate, but the burden of proof must be on the researcher to convince the IRB that adequate, timely funding is likely to come through.

Finally, the IRB should require that an underfunded study be held to several checkpoints following approval. For example, the study may need to be segmented into phases, each enrolling only as many participants as the researcher can afford at the time, until another tranche of funds becomes available. This ensures that enrolled participants can complete their participation in full and not be left blindsided by premature termination. In addition, the IRB should impose timelines to secure remaining funding. If reasonable deadlines are missed, the IRB should withdraw approval, minimizing further loss of resources and time.

Although underfunded studies should generally be rejected, these safeguards can provide some flexibility for exceptional circumstances. Because of the ethical issues associated with study noncompletion, however, the standard should be high and infrequently used.

## CONTINUING IRB OVERSIGHT

Before closing, it is important to address one additional type of funding insufficiency that IRBs may be faced with: studies that are fully funded at the time of approval but unexpectedly lose funding midstream. For example, the current Trump administration has cut research funding for several universities across the country and terminated hundreds of grants for specific studies, on a range of rationales including claims of inefficiency, failure to align with the administration's scientific priorities, or as punishment for alleged legal violations unrelated to the research.^24^ Together, these cuts have led to unprecedented levels of early study closures, affecting tens of thousands of study participants.^25^, ^26^ The dismantling of the US Agency for International Development has further contributed to the abrupt ending of clinical trials around the world, in many cases exposing participants to heightened risk.^12^ Although less extreme in scale, these concerns also arise in the private sector. For example, Swiss pharmaceutical company Roche recently terminated their clinical trial testing the efficacy of basmisanil, a medication under study to treat the rare genetic neurodevelopmental disorder Dup15q in children and adolescents, after seven children had enrolled and some in the US had been dosed with the experimental intervention. Roche explained the decision in financial terms, pointing to “trade‐offs made … to increase the overall portfolio value.”^27^

The removal of a study's funding in the middle of a trial due to changing government or business priorities is a serious violation of the ethical obligation to protect and respect participants.^28^ Although funding removal in cases like these is generally beyond an IRB's control, they should be aware of this issue and help institutions and investigators protect participants to the greatest extent possible. For example, several IRBs have adopted policies and offered guidance for investigators facing study termination due to recent loss of federal funding, with the goal of minimizing harm to participants.^29^ IRBs can help investigators develop action plans for safely winding down research and communicating with participants if bridge funding cannot be secured, including by offering templates for these plans and communications as well as generally acting as an outlet for questions and coordinated response. As an example of a relevant issue that investigators might not otherwise be aware of, IRBs can help inform them (and study participants) that loss of federal funding may terminate a study's automatic Certificate of Confidentiality, leaving data collected after that point at risk unless a discretionary certificate is secured.^29^ To make sure that IRBs are kept informed, researchers should be required to submit real‐time budget updates if there is a material change in a study's funding, along with plans to address budget shortfalls, akin to adverse event reporting. IRBs could also require researchers to certify that there have been no material funding changes during annual continuing reviews, as an added check.

Whenever possible, institutions should bolster their contractual relationships to prevent future rug‐pulls of funding from research. For example, institutions should seek to include specific contractual provisions with funders that condition study initiation on an agreement that the study will not be cancelled for financial reasons; they might also add terms imposing a financial penalty on parties that terminate a study early for reasons other than safety or effectiveness. Barring contractual provisions, funders at least should be asked to certify at the point of approval that they will not voluntarily terminate a study for financial reasons, which could help discourage the practice, even if legal enforceability is challenging.^17^ We recognize, however, that negotiations to implement such provisions will be limited by the bargaining power of any individual institution, especially for multisite research. If sponsors can easily find other willing sites without such protective clauses or requirements, they will likely do so. Accordingly, research institutions should consider collaboration and joint commitments to requiring standard funding protection provisions in their contracts, to the greatest extent possible in line with antitrust law. Institutional bargaining power may be especially limited for federal grants, the terms of which are typically “take it or leave it.” In these cases, institutions have little choice but to encourage the government to abide by its research funding commitments, as has been typical across prior administrations.

The problem of unexpected funding removal differs from that of anticipated funding shortages. Nonetheless, this issue is just as ethically problematic and warrants the implementation of all reasonable safeguards within the IRB's control.

## CONCLUSION

Given current review procedures, an IRB tasked with reviewing BrainStorm's anticipated trial would likely be unaware of whether the company had secured the funding necessary for its completion. To address the ethical issues associated with allowing underfunded studies to proceed, including failing to provide social value, leaving participants worse off, and exposing them to the completion misconception, IRBs should adopt procedures to ensure that budget and funding sufficiency are considered as part of protocol review. In most cases, studies will be adequately funded to support completion before they begin—but IRBs should also adopt procedures to guide decisions in exceptional cases, alongside adequate safeguards. IRBs take their responsibility for participant protection seriously. Attention to whether studies have the financial sufficiency to be completed is a critically important element of doing so.
